# Leveraging Artificial Intelligence to Improve the Diversity of Dermatological Skin Color Pathology: Protocol for an Algorithm Development and Validation Study

**DOI:** 10.2196/34896

**Published:** 2022-03-08

**Authors:** Eman Rezk, Mohamed Eltorki, Wael El-Dakhakhni

**Affiliations:** 1 School of Computational Science and Engineering McMaster University Hamilton, ON Canada; 2 Faculty of Health Sciences McMaster University Hamilton, ON Canada

**Keywords:** artificial intelligence, skin cancer, skin tone diversity, people of color, image blending, deep learning, classification, early diagnosis

## Abstract

**Background:**

The paucity of dark skin images in dermatological textbooks and atlases is a reflection of racial injustice in medicine. The underrepresentation of dark skin images makes diagnosing skin pathology in people of color challenging. For conditions such as skin cancer, in which early diagnosis makes a difference between life and death, people of color have worse prognoses and lower survival rates than people with lighter skin tones as a result of delayed or incorrect diagnoses. Recent advances in artificial intelligence, such as deep learning, offer a potential solution that can be achieved by diversifying the mostly light-skin image repositories through generating images for darker skin tones. Thus, facilitating the development of inclusive cancer early diagnosis systems that are trained and tested on diverse images that truly represent human skin tones.

**Objective:**

We aim to develop and evaluate an artificial intelligence–based skin cancer early detection system for all skin tones using clinical images.

**Methods:**

This study consists of four phases: (1) Publicly available skin image repositories will be analyzed to quantify the underrepresentation of darker skin tones, (2) Images will be generated for the underrepresented skin tones, (3) Generated images will be extensively evaluated for realism and disease presentation with quantitative image quality assessment as well as qualitative human expert and nonexpert ratings, and (4) The images will be utilized with available light-skin images to develop a robust skin cancer early detection model.

**Results:**

This study started in September 2020. The first phase of quantifying the underrepresentation of darker skin tones was completed in March 2021. The second phase of generating the images is in progress and will be completed by March 2022. The third phase is expected to be completed by May 2022, and the final phase is expected to be completed by September 2022.

**Conclusions:**

This work is the first step toward expanding skin tone diversity in existing image databases to address the current gap in the underrepresentation of darker skin tones. Once validated, the image bank will be a valuable resource that can potentially be utilized in physician education and in research applications. Furthermore, generated images are expected to improve the generalizability of skin cancer detection. When completed, the model will assist family physicians and general practitioners in evaluating skin lesion severity and in efficient triaging for referral to expert dermatologists. In addition, the model can assist dermatologists in diagnosing skin lesions.

**International Registered Report Identifier (IRRID):**

DERR1-10.2196/34896

## Introduction

### Background

Dermatology textbooks and atlases lack diversity in skin tones, which propagates structural racism in the health care system [1]. Descriptions and image documentations of differing dermatologic conditions have been largely based on light skin, posing challenges for dermatologists to promptly recognize conditions in darker skins [2]. These challenges may result in serious negative consequences when early diagnosis is crucial, such as in skin cancer. Prior work [3,4] has demonstrated that people of color have worse prognoses and lower survival rates attributed to delayed or incorrect diagnoses. In people of color, squamous cell carcinoma is the most common type of cancer, and delayed diagnosis is linked to higher rates of metastasis and a decrease in the 10-year survival rate to 20% [4]. The second most common cancer is basal cell carcinoma, in which 50% of the cases are pigmented and often misdiagnosed as melanoma, seborrheic keratosis or nevus sebaceous [4]. Finally, people of color are typically diagnosed at more advanced stages of melanoma, which is responsible for 75% of mortality from all skin cancers [5], and this late diagnosis causes a dramatic decrease in the 5-year survival rate to 66.7% compared to 92.5% in individuals with light skin [6].

### Artificial Intelligence in Dermatology

Artificial intelligence refers to techniques that allow machines to mimic human behavior to analyze complex data [7]. Deep learning is a leading technology in artificial intelligence that leverages the capabilities of neural networks to analyze complex system structures independently from human intervention. As a result, deep learning has led to breakthroughs in the development of intelligent medical image analysis and diagnosis with performance comparable to that of health care providers [8].

In dermatology, deep learning models have been performing on par with dermatologists in diagnosing skin cancer. In melanoma classification, a convolutional neural network trained on 12,378 dermoscopic images and tested on 100 clinical images performed on par with 145 dermatologists [9]. The convolutional neural network and dermatologists achieved a mean sensitivity of 89.4%, while the convolutional neural network had a specificity of 68.2% compared with a sensitivity of 64.4% for the dermatologists. Furthermore, a convolutional neural network was utilized to assist 12 board-certified dermatologists in melanoma diagnosis [10]. With the support of the convolutional neural network, the mean sensitivity of the dermatologists significantly (*P*=.003) improved from 59.4% to 74.6%, and the mean accuracy significantly (*P*=.002) increased from 65.0% to 73.6%.

Haenssle et al [11] compared the performance of a deep learning–based model to that of 96 dermatologists with varying levels of experience in classifying skin lesion dermoscopic images as malignant or benign. The model obtained a sensitivity of 95.0% (95% CI 83.5%-98.6%) and a specificity of 76.7% (95% CI 64.6%-85.6%); however, the dermatologists had a mean sensitivity of 89.0% (95% CI 87.4%-90.6%) and specificity of 80.7% (95% CI 78.8%-82.6%). In another experiment [11] that involved diagnosis using a combination of dermoscopic images and clinical vignettes, dermatologists had a sensitivity of 94.1% (95% CI 93.1%-95.1%) and a specificity of 80.4% (95% CI 78.4%-82.4%). At the same specificity level as the dermatologists, the deep learning–based model had a sensitivity of 95% (95% CI 83.5%-98.6%) [11].

In diagnosing nonpigmented skin cancer [12], the performance of a convolutional neural network trained on 13,724 images (7895 dermoscopic and 5829 clinical) and tested on 2072 images was compared with that of beginner, intermediate, and expert dermatologists (95 categorized by years of experience) and achieved an area under the curve (AUC) of 0.742 (95% CI 0.729-0.755) compared with an AUC of 0.695 (95% CI 0.676-0.713) for the dermatologists. For particularly challenging conditions, 37.6% (95% CI 36.6%-38.4%) of the network’s diagnoses were correct, which was higher than the corresponding percentages for beginner and intermediate dermatologists but less than that of expert dermatologists (accuracy 40.0%, 95% CI 37.0%-43.0%). Given the low number of expert dermatologists in many health care jurisdictions [13], this technology would be very useful in assisting nonexpert dermatologists (eg, general practitioners) in triaging skin lesions that need a referral to an expert dermatologist for advanced assessment.

Unfortunately, despite these advancements, the ability to take full advantage of deep learning capabilities is limited due to the lack of adequate quantity and quality of real-world data. Furthermore, and notwithstanding its advantages, the use of deep learning has further put people with darker skin tones at a disadvantage, because training data used in the development of published models lack the true breadth of human skin tones [14,15]. The paucity of nonwhite skin tones in training data limits the generalizability of developed models to nonwhite skin tones. For example, a deep learning–based classifier to diagnose 12 malignant and benign skin lesions using clinical images [16] trained on Asian skin images and validated with Caucasian images classified basal cell carcinoma with an AUC of 0.78, SD 0.02. However, when the training data was augmented by including Caucasian images, the AUC improved to 0.90, SD 0.01. Despite of the excellent performance metrics during development and validation, the model’s generalizability was deficient when tested on a different patient population [17].

The lack of skin tone diversity also limits the study of the relationship between skin tone and diagnostic accuracy. Analysis performed on 2 publicly available data sets—the international skin imaging collaboration (ISIC), which consists of 10,015 dermoscopic images from 7 skin diseases data set, and SD-198, which consists of 6548 clinical images from 198 skin diseases [18]—showed no measurable correlation between classification accuracy and skin tone, which was attributed to the fact that the images in the data sets consisted mainly of light skin tones [18].

### Study Goals

Several promising initiatives are being employed to address the lack of diversity, such as a deliberate focus on increasing the diversity of students in medical schools and postgraduate dermatology training programs [19,20], emerging textbooks and literature targeting darker skin pathology [21], and new websites to build a database of skin pathology in darker skin tones [1]; however, these efforts are complex, challenging, and take time, thus, implementing a rapid yet effective solution to address this gap is imperative.

We aim to (1) identify the underrepresented tones in publicly available dermatology clinical image atlases; (2) generate realistic images for darker skin with closely related malignant and benign conditions; (3) extensively evaluate the images, using quantitative ratings as well as qualitative human expert and nonexpert ratings; and (4) develop a classification model using several deep learning networks to detect malignancy on all skin tones.

## Methods

### Overview

**Figure 1:**
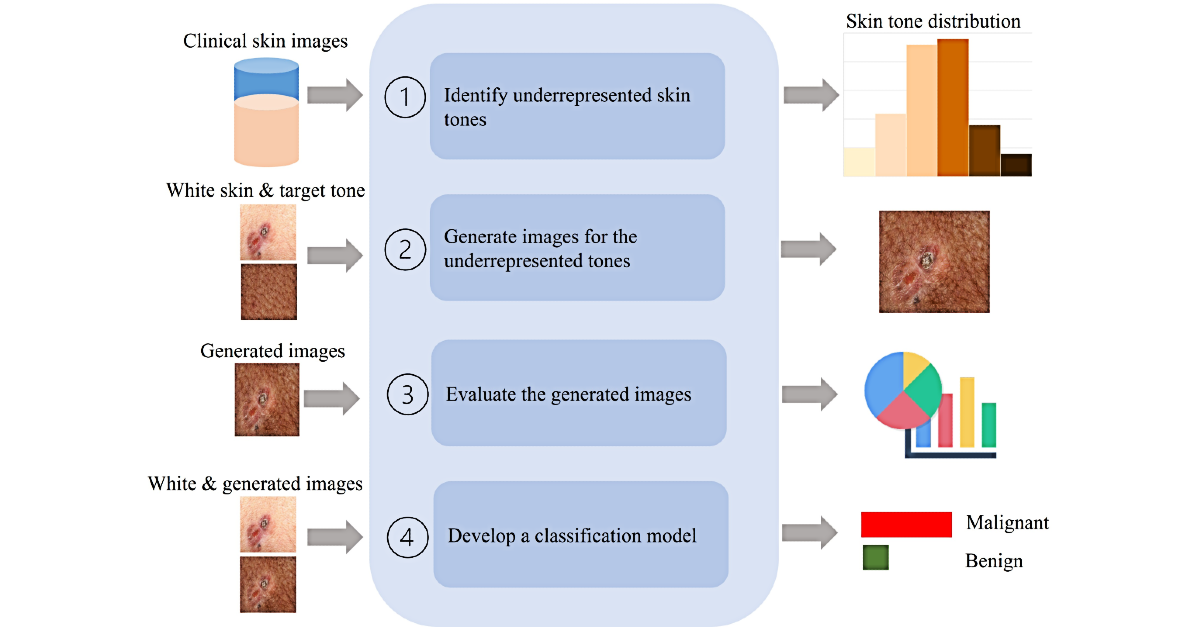
Phases of the proposed work.

This work has 4 main phases (Figure 1). The focus of this work is on common malignant skin pathology and closely related disorders that resemble those malignant lesions and form part of their differential diagnosis; therefore, we collected all clinical images representing those conditions such as basal cell carcinoma, squamous cell carcinoma, melanoma, and nevus from DermNet NZ (994 images) [22], ISIC 2018 (100 images) [23], and a dermatology atlas (607 images) [24]. Images from ISIC and DermNet NZ (Set A) were utilized in training, finetuning, and internal validation. The dermatology atlas images (Set B) will be utilized only for testing the classification model in phase 4 (Table 1).

**Table 1:** Image distribution for training, finetuning, and internal validation (Set A) and for testing (Set B).

Class	Set A (n=1094), n (%)	Set B (n=607), n (%)
Malignant	634 (58)	508 (83.7)
Benign	460 (42)	99 (16.3)

### Phase 1: Underrepresented Skin Tones Identification

**Figure 2:**
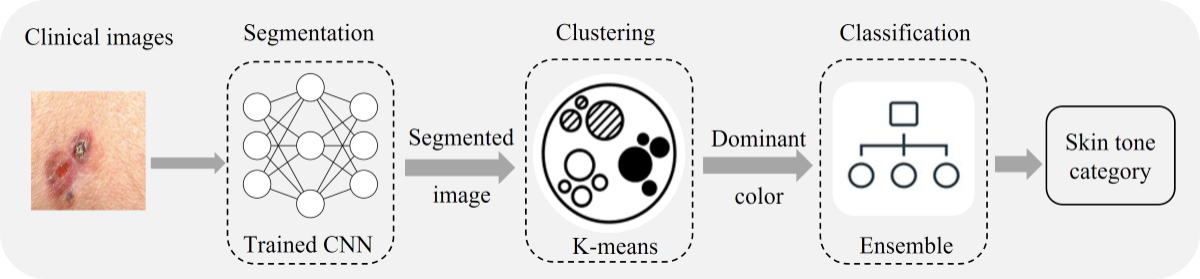
Modules of phase 1. CNN: convolutional neural network.

The goal of this phase (Figure 2) is to analyze the images to determine the skin tone distribution and quantify the underrepresentation of the darker tones in the data sets.

#### Skin Image Augmented Segmentation

**Figure 3:**
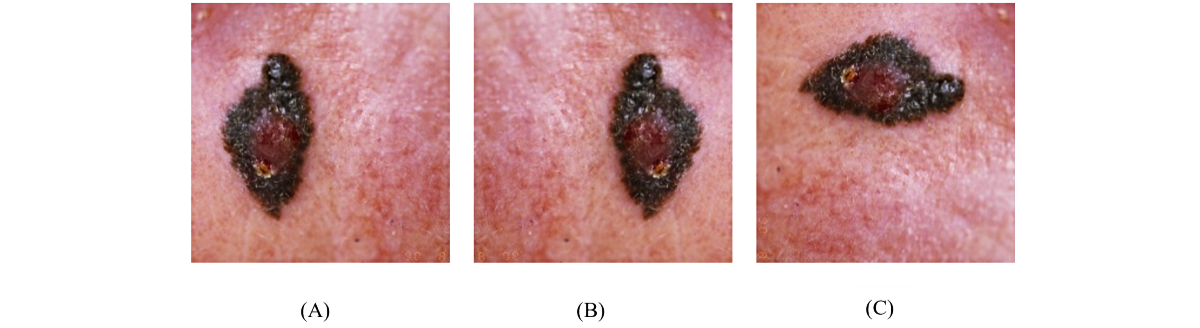
Data augmentation on skin image (A) original image (B) horizontal flipping (C) 90° rotation.

Image data augmentation is the process of applying transformations on the images such as flipping, cropping, and rotating to increase the training set size, improve training data variance, and reduce overfitting [25]. A set of 500 clinical images was randomly selected from Set A; images were horizontally flipped and rotated by 90° to yield additional images for the data set (Figure 3).

The images (500 original and 1000 augmented) were utilized to train a segmentation network to separate the disease region from the underlying skin and allow the analysis of the skin color. Trained segmentation networks can be used to improve skin image classification [26]. A deep learning–based image segmentation network developed by Azad et al [27] was employed due to its high accuracy in segmenting dermoscopic skin images. The network was initially trained on 2594 dermoscopic skin images [27]. We adapted the network to segment clinical skin images through transfer learning, by performing an additional cycle of training on the original and augmented images (1500). The training data were randomly split into 1200 (80%) images for training, 150 (10%) images for validation, and 150 (10%) images for testing. Because segmentation classifies each pixel in the image as disease or skin, it can be considered a binary classification problem; therefore, the segmentation model was assessed using accuracy, sensitivity, specificity, AUC, and Jaccard similarity.

#### Skin Image Clustering

**Figure 4:**
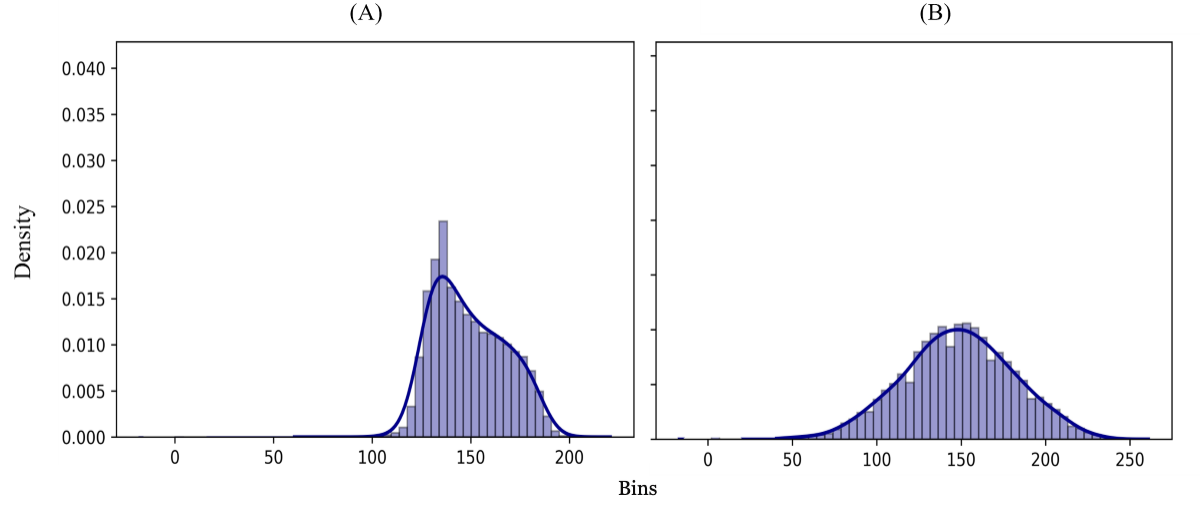
Skin image histogram (A) before and (B) after equalization.

The segmented normal skin regions in the images were preprocessed to detect and remove any possible nonsegmented disease pixels resulting from a variation of the skin color, improve the quality of the images, and allow for accurate skin tone analyses. The contrast of the images was enhanced (Figure 4) using the contrast-limited adaptive histogram equalization, which has been widely applied in medical image enhancement, such as retinal fundus images [28], breast mammography [29], and bone fracture images [29]. The contrast enhancement algorithm divides the image into sections and creates a histogram for each section that is utilized to redistribute the brightness across the image. This method has outperformed other contrast enhancement methods because it limits the amplification of the contrast across the image and hence reduces noise [30].

**Figure 5:**
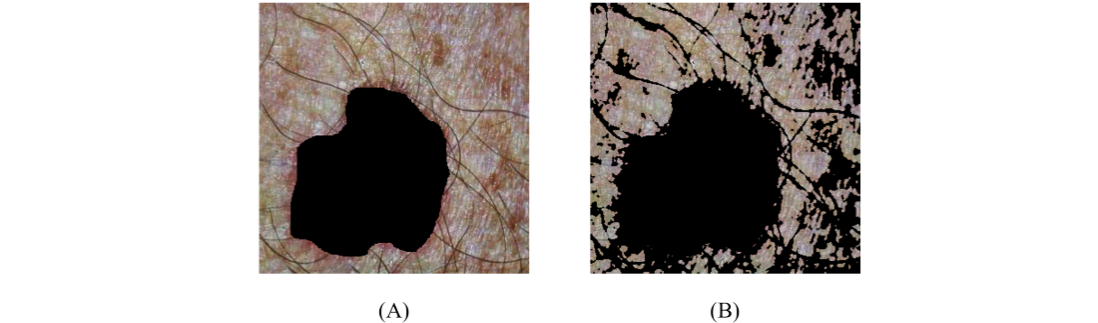
(A) Original and (B) contrast-adjusted (with thresholding) image.

Applying contrast adjustment helped to identify pigmented spots and made them easier to remove. A thresholding technique (Figure 5) that analyzed the updated image histogram to classify each pixel as foreground or background [31] was implemented to detect and remove any objects on the skin such as colored spots and hair. As a result, the image included only the pixels that truly reflected skin color.

Processed skin pixels were subsequently analyzed to determine the dominant skin tone using k-means clustering [32] to group the pixels based on color values. The number of clusters was selected to minimize the sum of squared errors and improve in-cluster cohesion [33]. The cluster that had the maximum number of pixels was considered the dominant cluster, and its center was considered the dominant color.

#### Skin Tone Classification

Although skin tone is easy to perceive, it is challenging to evaluate quantitively. Fitzpatrick [34] defined 6 skin color categories (very light, light, intermediate, tan, brown, and dark). The Melanin Index is one of the most reliable metrics to quantify human skin color based on skin reflectance (using a reflectance spectrophotometer) [35]. However, the use of the melanin index is limited to dermatologists as it requires specialized equipment. Individual typology angle is another metric to evaluate the skin tone category in which the skin color is utilized to calculate an angle that can be assigned to a skin category [36]. Unfortunately, the former skin categorization approach has exhibited inconsistencies and inaccuracies compared to the perceived skin tones [18].

**Figure 6:**
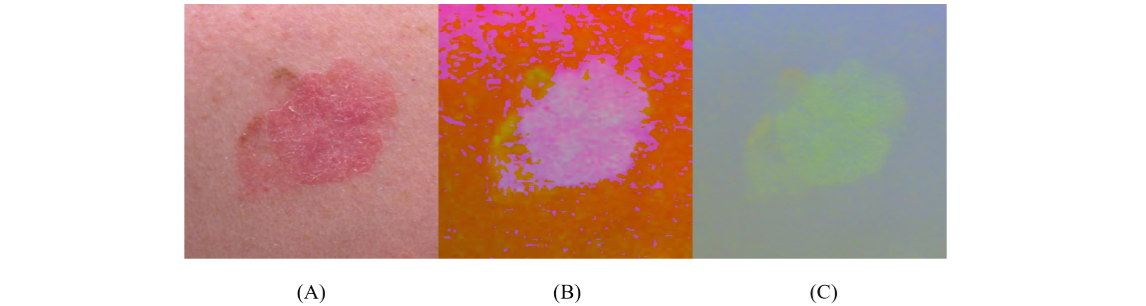
(A) RGB (red, green, blue); (B) HSV (hue, saturation, value); and (C) Lab (luminance, red–green, blue–yellow) color spaces.

We developed a skin categorization model that classified a dominant skin color into a skin category. An ensemble model, which included k-nearest neighbor [37], random forest [38], and naïve Bayes [39] methods, was implemented to classify the dominant skin color as very light, light, intermediate, tan, brown, or black. For training and validation, a set of 100 skin color variations, represented in the RGB (red, green, blue) color space, was collected from the human skin color database [40]. RGB features were processed to create supplementary color features from different color spaces such as HSV (hue, saturation, value) and Lab (L represents luminance, *a* represents the range from red to green, and b represents the range from blue to yellow) [41] to provide the model with sufficient color information (Figure 6). The model was tested on the dominant colors extracted from the clustering step and evaluated using accuracy and AUC.

### Phase 2: Image Generations for Underrepresented Tones

Style transfer and deep blending image generation methods will be investigated. Both methods are based on image feature extraction and blending using the Visual Geometry Group network trained on the ImageNet database with millions of images for object localization and recognition [42]. Given that convolutional neural networks trained using sufficient labeled data on object recognition are capable of extracting high-level feature representations regardless of the data set [43], style transfer and deep blending can be generalized to the skin image generation problem.

**Figure 7:**
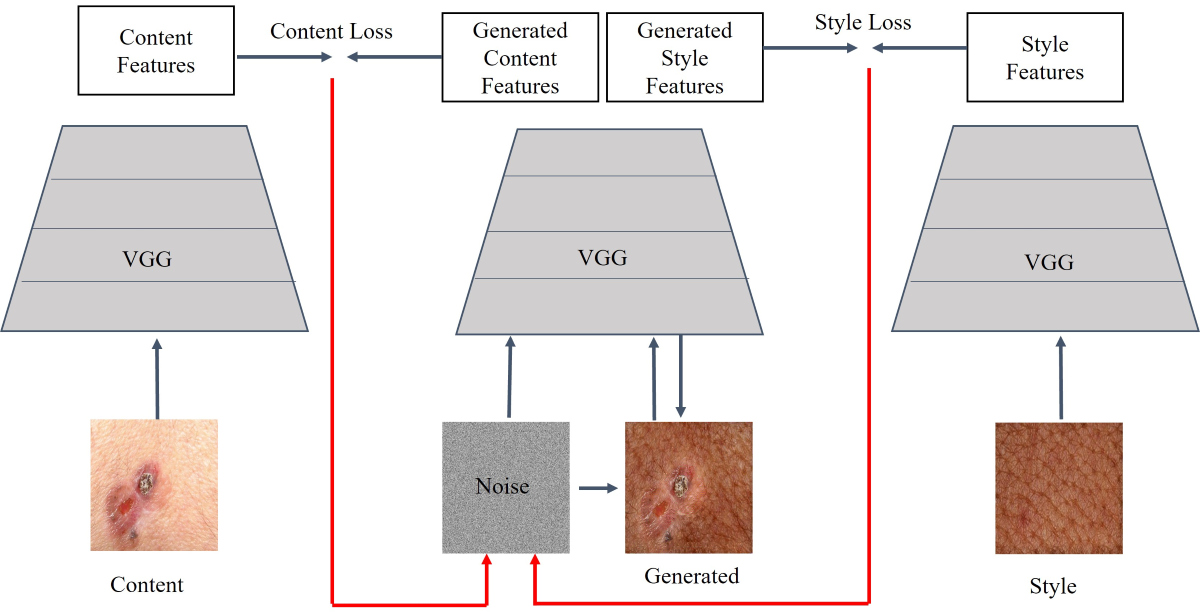
Style transfer procedure. VGG: Visual Geometry Group.

Style transfer has been mainly applied to create stylized artwork [44]. This method will be used to generate skin images with dark skin tones by extracting the features of (1) a content image containing the skin pathological condition and (2) a style image with the target skin color. A new image containing a weighted blend of both feature sets will then be generated starting from noise and iteratively improving by minimizing the content and style loss (Figure 7).

**Figure 8:**
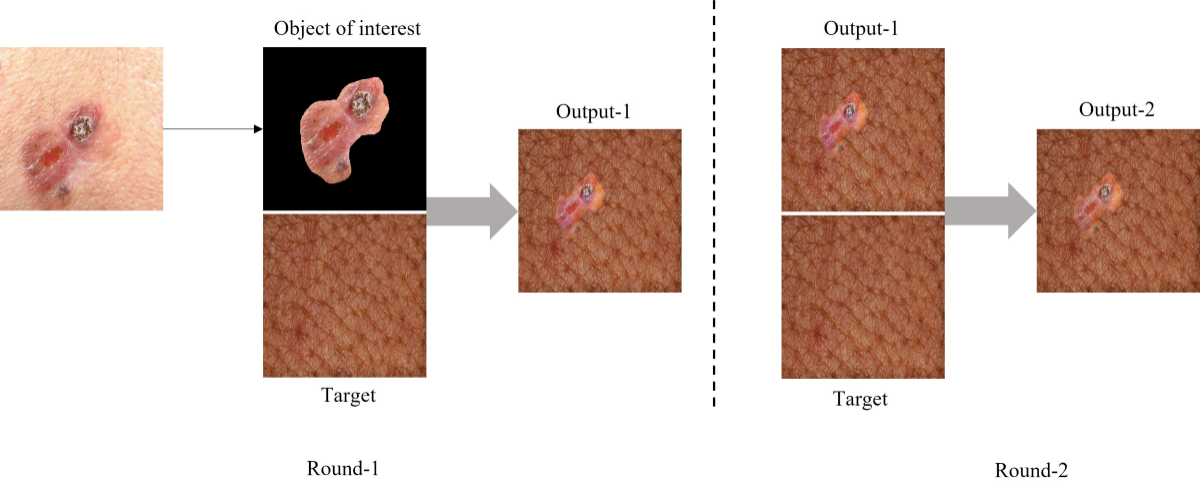
Deep blending rounds.

Deep blending has been used to blend an object with a target background image technically, as in style transfer; however deep blending has 3 main differences [45]: only the object of interest in the source (content) image is blended with the target (style) image, thus it requires a segmentation mask, a new loss metric is added to the content and style loss to minimize the sharp intensity change between the source object and target image, and 2 rounds of blending are performed—one with the source object and target image, and the second with the output image of the first round and the target image (Figure 8). We will use this method to (1) achieve a seamless blending of the content disease region and the target skin features and (2) use the image generated from the first step with the style image as input to the network to impose the target skin features (eg, color and texture) on the disease region and the blending boundary to provide a smooth realistic image.

The parameters of style transfer and deep blending methods, such as the number of network layers, the content to style weights, and the degree of lesion pigmentation, will be finetuned. The methods will be employed to generate images for underrepresented skin tones (based on the findings of phase 1).

### Phase 3: Generated Image Evaluations

#### Overview

The images that are generated will be assessed quantitively and qualitatively. Primarily image realism and disease presentation will be evaluated through numerical image quality metrics as well as human expert and nonexpert rating. The best performing image generation technique will be utilized to generate diverse images to train the classification model.

#### Quantitative Evaluation

Quantitative evaluation of the generated images will be performed using 2 image quality assessment metrics—the blind reference-less image spatial quality evaluator, a reference-less metric that quantifies the loss of image realism in the presence of distortions by extracting 18 statistical features to assign a quality score with a support vector machine regressor [46], and the structural similarity index measure, which compares the structure, texture, and edges of a reference image (the original image) with a modified image and provides a similarity score [47].

#### Qualitative Evaluation

The human visual Turing test, wherein participants are asked to classify images as real or generated, will be conducted. Participants of this test will be medical personnel with varying experience and nonmedical personnel. The classification accuracy, false positive rate (the ratio of generated images classified as real), and true positive rate ( the ratio of real images classified as real) will be calculated. Furthermore, a regression model will be implemented to study the significance of the participants’ background in distinguishing the generated images.

Disease identification will also be conducted, which will include solely dermatologists with different years of experience as participants, to evaluate the accuracy of disease presentation in the generated images. The dermatologists will be asked to choose the disease that best describes a set of real and generated images with various malignant and benign conditions. The rate of correctly identified images will be calculated for each disease, image group (real or generated), and skin tone. In addition, disease misdiagnosis rates will be compared with that in published literature pertaining to misdiagnoses in skin of color.

### Phase 4: Classification Model Development

The goal of this phase is to develop a malignancy detection model using real and generated diverse images. To develop the model, skin images from Set A and dark skin images generated in phase 2 will be utilized for training and validation. Set B will be used as an independent test set. Set A contains primarily images with light skin (collected from New Zealand). Set B was collected from Brazil where there are varying skin tones (based on Fitzpatrick classification) in the population compared with that of New Zealand [48]. The diversity in Set B, which was confirmed in phase 1, will allow the generalizability of the model to darker skin tones to be evaluated and the impact of the generated images on the classification accuracy to be determined.

Several classification networks will be trained and validated on Set A after handling data skewness. Data augmentation techniques will be utilized to balance class distribution and ensure that the model is not biased toward any class. Given that the developed model will be well balanced, no augmentation on Set B will be performed during the testing phase.

As training deep learning–based classification networks requires large data sets, adapting pretrained networks is important to make use of the network’s calculated weights instead of starting from random weights which requires more training data. Transferring knowledge from networks pretrained on a large number of images, then enriching that knowledge to classify skin images helps to overcome the lack of data. Therefore, deep learning network architectures such as GoogLeNet [49] and ResNet [50], initially trained with millions of natural images from the ImageNet data set, will be adapted. Existing weights obtained from pretraining will be customized to fit skin image classification.

The classification process will follow 2 approaches to evaluate the effect of the generated images on skin tone diversity, classification accuracy, and generalizability. (1) The convolutional neural networks will be trained on 80% of Set A (randomly selected) and their corresponding generated darker color images. The remaining 20% will be used for validation while building the model and to update the network weights. This approach will help increase the number of training instances and is expected to familiarize the network with diverse human skin tones. (2) The convolutional neural networks will be trained on the same 80% of Set A images and their corresponding augmented images. The remaining 20% will be utilized for validation, thus the training set and the validation set in both approaches have the same sizes and same original images. In both approaches, Set B will be utilized for testing.

Accuracy boosting will be attempted by integrating supplementary information as separate features, such as skin color category, lesion anatomic distribution, and lesion textual description (lesion color, shape, texture, clinical presentation, associated conditions such as scarring and inflammation). For example, in people of color, pigmented basal cell carcinoma is more prevalent, which will be captured by the generated images, however, some features, such as the solitary papule appearance, will be provided as textual description [51] to address missed appearance factors in the generated images, which will improve malignancy detection in the skin of color where lesions might look different or can be associated with unusual signs [51].

The models will be evaluated using accuracy and AUC. We will compare model classification performance to that of the dermatologists and report the dermatologists’ diagnosis performance with the aid of the developed model as a second opinion. In addition, the correlation between performance measures and skin tone will be calculated.

## Results

Phase 1 was initiated in September 2020 and completed in March 2021. Phase 2 was subsequently initiated and will be completed in March 2022. Phase 3 and phase 4 will be conducted in parallel; the study is expected to be completed by September 2022.

**Figure 9:**
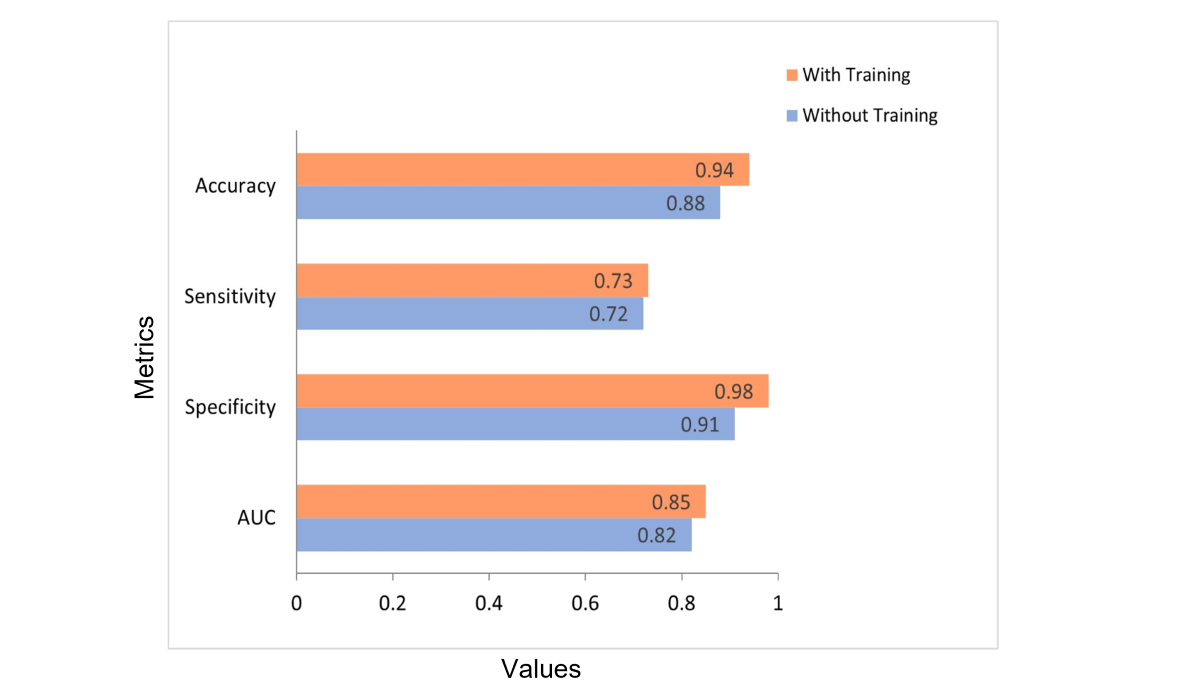
Performance measures of the segmentation before and after training.

In Figure 9, a comparison between the segmentation model with and without training on clinical skin images shows that training improves all performance metrics. Accuracy increased from 0.88 (95% CI 0.8798-0.8802) to 0.94 (95% CI 0.9399-0.9401), sensitivity slightly increased from 0.72 (95% CI 0.7197-0.7203) to 0.73 (95% CI 0.7297-0.7303), specificity significantly increased from 0.91 (95% CI 0.9098-0.9102) to 0.98 (95% CI 0.9799-0.9801), AUC increased from 0.82 (95% CI 0.8155-0.8162) to 0.85 (95% CI 0.8538-0.8544), and Jaccard similarity increased from 0.88 (95% CI 0.8798-0.8802) to 0.94 (95% CI 0.9399-0.9401).

**Figure 10:**
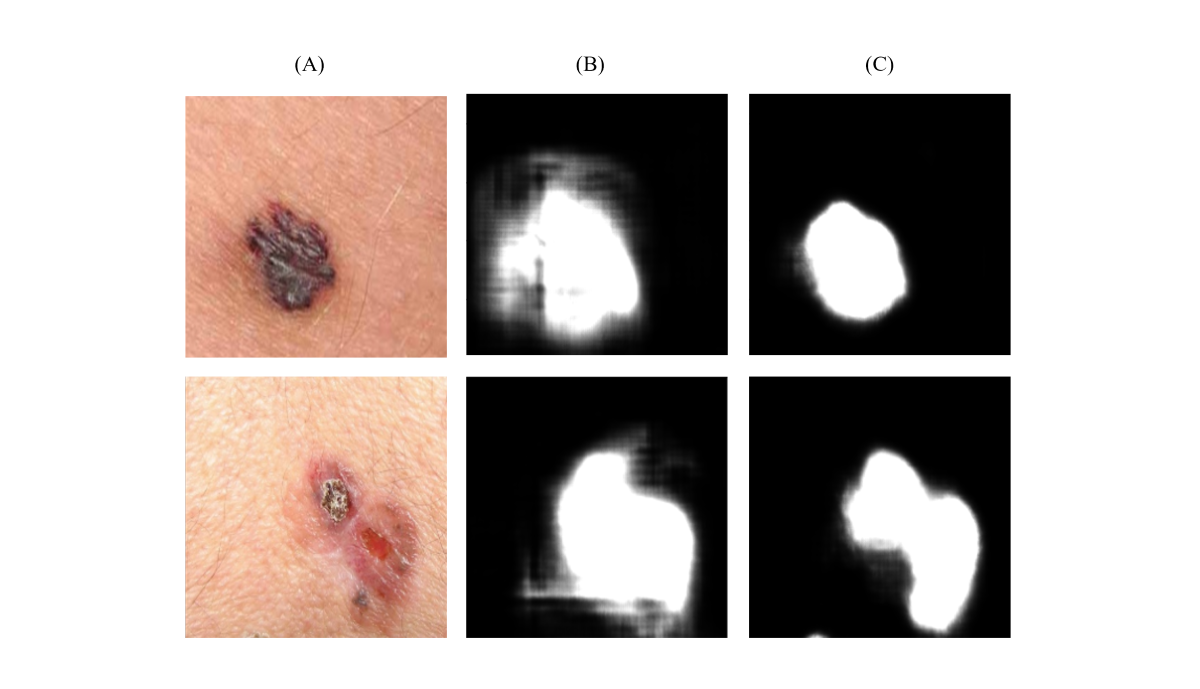
(A) input; (B) without training; and (C) with training.

**Figure 11:**
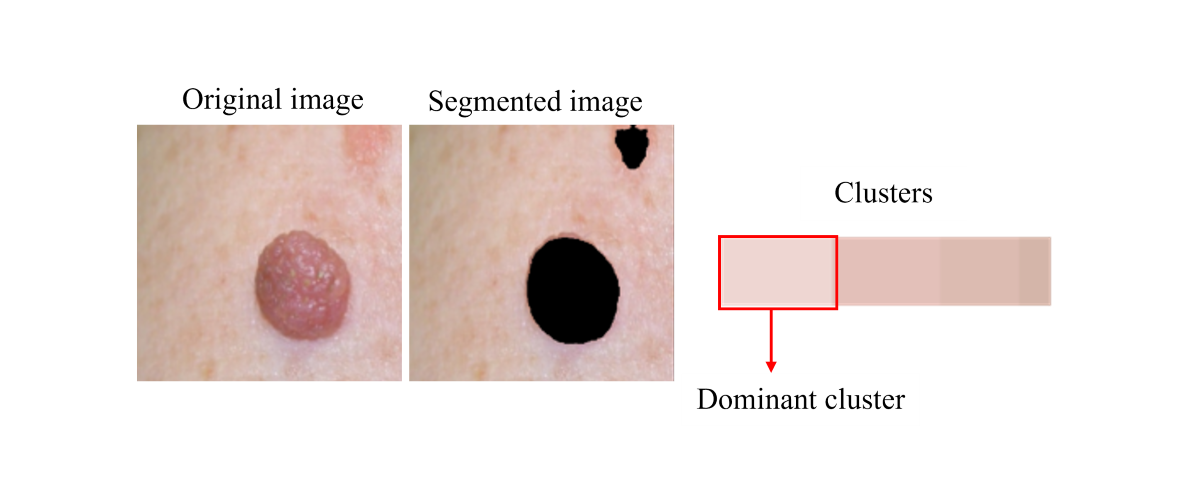
Clustering results.

**Figure 12:**
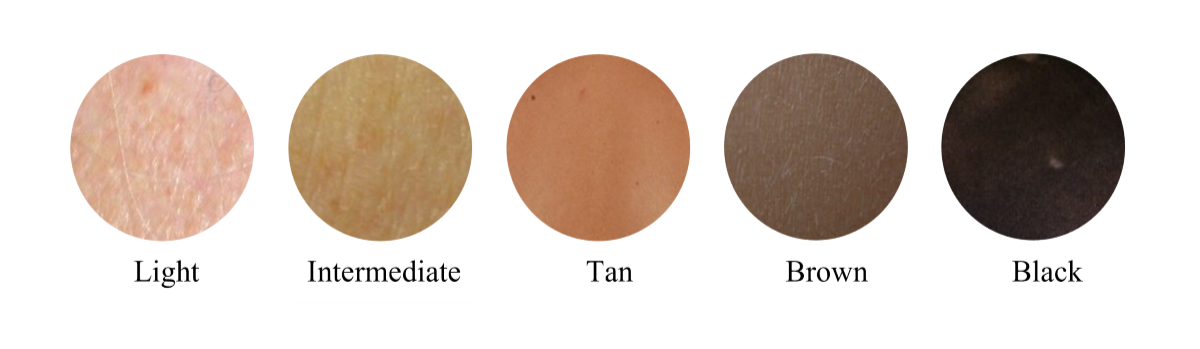
Skin tone categorization results.

Segmentation masks that outlined the region of the disease improved with training (Figure 10). After image enhancement and thresholding, the bar of clusters’ center color is plotted; the first cluster is the largest and dominant one (Figure 11). In classifying the dominant colors into skin tone categories (Figure 12), the model performed well (accuracy 0.95, 95% CI 0.86-1.0; AUC 0.98, 95% CI 0.92-1.0).

**Figure 13:**
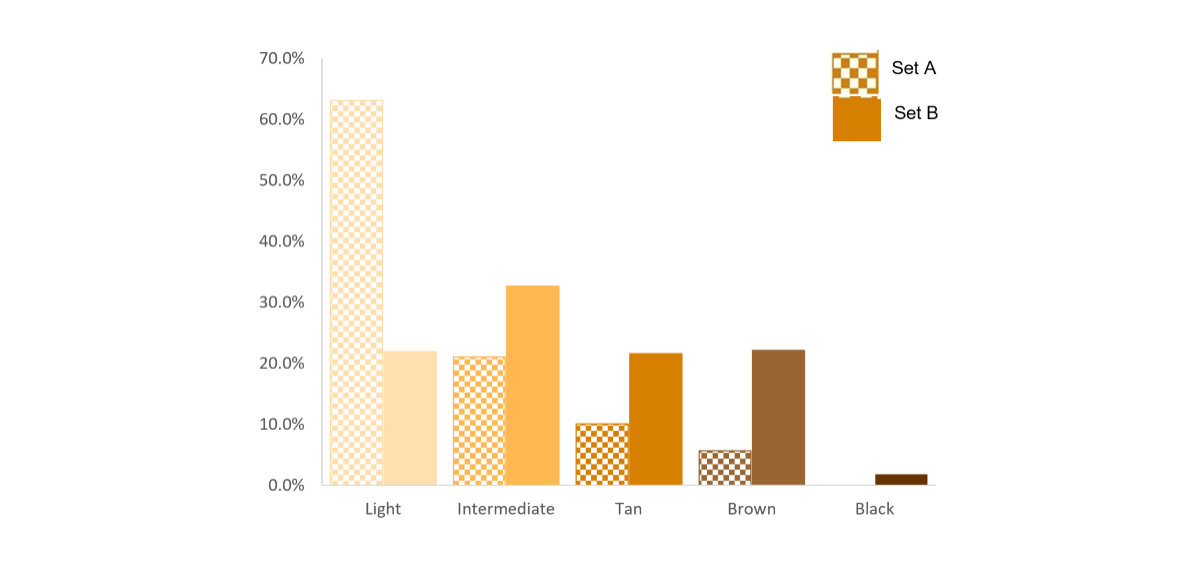
Skin tone distribution in set A and set B.

In Set A, more than 60% of the images were light skin, 20% were intermediate, and only 20% were tan, brown, and black; in Set B, 45.5% of the images were tan, brown, and black (Figure 13).

## Discussion

### General

Currently, we have successfully completed the first phase and the second phase is in progress. Based on the results of phase 1, phase 2 will be configured to generate images for the underrepresented skin tones in Set A, thus a diverse image bank will be created. Phase 3 when completed will extensively evaluate the generated images to ensure their quality and high disease presentation. Since an image library that reflects the true breadth of human skin tones will become available, we will build generalizable deep learning models to detect malignancy. Moreover, the detection accuracy will be boosted by employing supplementary features extracted from disease clinical presentation and anatomic distribution. Furthermore, the correlation between classification accuracy and skin tones can be studied, and the performance of dermatologists with the aid of the skin cancer early detection system will be assessed in phase 4.

The development of the integrated artificial intelligence-based skin cancer early detection system for all skin tones incorporates 4 main milestones: identifying underrepresented skin tones, generating a diverse clinical image bank for various malignant and benign conditions, broadly evaluating the generated images, and developing a generalizable classifier to detect malignancy in any skin tone.

The system is designed to analyze clinical images to increase its usability as digital cameras are easily accessible. The system will advantage all skin tones, consequently increasing the dark skin tone inclusion. The system is also expected to raise the clinical index of suspicion and boost the detection rates of malignant lesions. Finally, the system will assist prioritize patients’ referrals to expert dermatologists, for faster diagnosis and help dermatologists with malignancy detection.

The preliminary findings show that the segmentation components demonstrate high accuracy and the quality of the pilot-generated images is promising.

### Comparison With Prior Work

Image generation using deep learning had been applied to improve pathology diversity and balance the data. Generative adversarial networks have been utilized in a prior study [52] to generate realistic dermoscopic skin images for various malignant and benign conditions to overcome data skewness. Unlike clinical images, dermoscopic images are captured while zooming in to focus on the disease, thus skin tone was not a factor.

Generative adversarial networks have been also utilized to generate clinical skin images for 8 skin conditions (eg, skin tag and melanoma). In [53], a semantic map encoding each input image was manually generated and given as part of the input while training, testing, and generating images, which significantly limited the system’s applicability. Although it was possible to generate images with different skin tones using semantic maps, there was no focus on the darker skin tones. In addition, the ratio of generated images detected as real was relatively low (0.30) and the ratio of correctly identifying skin disease was 0.45. Moreover, the number of participants involved in image quality assessment was insufficient to draw significant conclusions.

To the best of our knowledge, to date, no published study has focused on generating clinical images that reflect the diversity of skin tones. Accompanied by the etiological factors and the anatomic distribution of skin cancer in people of color, the images that are generated will be a valuable resource that can be utilized in education and research, in addition, to its use in this study.

### Limitations

The proposed system has limitations, the quality of the original images is vital in generating high-quality images. The system depends on publicly available data sets, thus there are data availability limitations; however, we will overcome this limitation by implementing data-efficient and pretrained deep learning methods. Compared with the real images of darker skin tones, generated images might not include all factors that can affect the appearance of the skin lesions on people of color. We will mitigate this issue by reflecting the pigmentation on the lesion consistently with the skin color, and the classification model will be supplemented with textual features detailing key clinical presentations of different lesions on darker skin tones.

### Implications

Until a real-image repository collected from patients with diverse skin tones becomes available, this study is the first step to filling the gap regarding skin tone diversity toward the goal of achieving racial equity in dermatology diagnosis. The generated image bank will increase the inclusion of dark skin images in dermatology research, the study will leverage the capabilities of deep learning–based cancer detection methods using these images. Our model can be trained in the future with real images of dark skin once dermatology atlases with a large number of dark skin pathologies become available.

## Abbreviations

AUC: area under the curve
ISIC: International Skin Imaging Collaboration
RGB: red, green, blue color space
